# Shared decision making from reintegration professionals’ perspectives to support return to work: a qualitative study

**DOI:** 10.1186/s12889-021-10365-z

**Published:** 2021-02-09

**Authors:** Marloes Vooijs, Nicole M. C. van Kesteren, Astrid M. Hazelzet, Wilma Otten

**Affiliations:** Netherlands Organisation for Applied Scientific Research TNO, Sustainable Productivity and Employability, Leiden, The Netherlands

**Keywords:** Shared decision making, Patient participation, Autonomy, Evidence based, Employment, Employability, Return to work

## Abstract

**Background:**

Work participation is an important determinant of public health; being unemployed leads to a decrease in an individual’s health. In the Netherlands, people with a work disability can apply for disability benefits, in which people also receive support to return to work (RTW). A method, currently used in the medical sector, that can include both the perspective of the reintegration professional and of the individual in the process of RTW, is shared decision making (SDM). In this article we explore to what extent reintegration professionals currently use SDM, and to what extent they prefer to use SDM in their ideal interaction with clients.

**Methods:**

We performed semi-structured interviews with fourteen reintegration professionals from four different municipalities. The transcripts were coded according to content analysis, applying open and axial coding.

**Results:**

Reintegration professionals emphasised the importance of having a good relationship with clients, of building trust and collaborating as a team. They did not inform their clients that they could be part of the decision-making process, or discussed a shared goal. Although professionals did emphasise the importance of aligning their approach with the preferences of the client and though they tried to offer some choice options, they did not mention available options, discussed the pros and cons of these options or evaluated decisions with their clients. Furthermore, they did not mention any of these aspects in their ideal interaction with clients.

**Conclusions:**

SDM has a potential value, because all professionals underline the importance of having an alliance with clients, collaborating as a team, and striving to align their approach with the preferences of the client. However, professionals currently perform a limited set of SDM steps. Additional knowledge and skills are needed for both reintegration professionals and municipalities so that professionals can consider and reflect on the value of using SDM, or SDM steps, in supporting RTW. Providing clients with knowledge and skills seems necessary to facilitate both self-management and SDM.

**Supplementary Information:**

The online version contains supplementary material available at 10.1186/s12889-021-10365-z.

## Background

Although work participation is an important determinant of public health, many people are confronted with a work disability [1–3]. People with a work disability face unemployment twice as much as people without a disability [4, 5]. Unemployment leads to a decrease in health for these individuals, since work provides structure, social contacts, a sense of belonging and the feeling of being part of society [6–8]. In addition, unemployment greatly reduces the labour supply, which limits economic growth for society as a whole [9].

In the Netherlands, people with a work disability can apply for disability benefits. The work disability policy is decentralised and delegated to the municipalities in order to provide individuals, referred to as ‘clients’, with customised support [10]. In recent years, most municipalities have shifted towards a more support-oriented policy instead of a more control-oriented policy, because it proved to be more effective in supporting individuals to return to work (RTW) [11, 12]. To support clients, that is people who receive a disability benefit and being supported towards RTW, many municipalities have pre-purchased re-integration interventions executed by a re-integration organisation, such as schooling or training (e.g., learning Dutch language), empowerment interventions, work placements to ‘learn’ the client to work etc., which are deployed depending on a client’s situation [13].

After the application for a disability benefit has been received, reintegration professionals in the work and income domain check and approve the issuance of this disability benefit (‘legal aspect’) and primarily support the client toward RTW (‘goal-oriented’), adapted to his or her abilities [14]. Depending on the municipality, these functions are either executed by one reintegration professional so that he/she has the complete overview of a client’s situation, or intentionally distributed among two reintegration professionals as some municipalities strongly believe that dividing the goal-oriented and the legal aspect is more effective in building an alliance with a client [15].

The decisions and actions in order for a client to RTW, like promoting an individual’s employability, greatly impact a client’s life. Therefore, it is important to include the client’s preferences in the decision-making process [15–17]. A method to stimulate inclusion of the perspectives of both the professional and the client, is shared decision making (SDM) [15]. SDM originates from the medical sector and focuses on the three-talk model that contains three phases: ‘team talk’, ‘option talk’ and ‘decision talk’ [18]. Firstly, in team talk a professional and the patient must cooperate as a team, after which the patient needs to be informed that he or she can be part of the decision-making process, and the professional and the patient need to discuss the shared goal they want to achieve [18]. Secondly, the patient is informed about all the available options to reach the shared goal and all the possible pros and cons of these options are discussed, that is option talk [18]. Finally, in decision talk, the preferences for the various options are discussed and a shared decision is made, which could also entail delegating the decision to the professional [18].

The premise of SDM is that both the physician and the patient have unique and valuable information relevant to the decision; the physician provides evidence- and experience-based knowledge, while the patient contributes his or her preferences and personal experiences [18, 19]. The use of SDM in doctor-patient relationships results in greater satisfaction with the alliance between physician and patient, higher levels of self-management and autonomy, and greater compliance with the plan of action [16]; outcomes which could highly facilitate the process of RTW. Therefore, in this article we want to explore the research question whether reintegration professionals already use one or more of the SDM steps in their interaction with clients and to what extent, and whether they would like to use one or more of these steps in their ideal interaction with clients in the future.

## Methods

We performed semi-structured interviews with reintegration professionals working for municipalities. Before the start of the interviews, we developed an interview guide to answer the research question of this study (see Supplementary Material), based on the three-talk model explaining SDM [18] and multiple work sessions with the research team to formulate and structure the questions. Features of the consolidated criteria for reported qualitative research (COREQ) [20] were used to improve the design and quality of reporting the present qualitative research and are addressed in this methods section (see Supplementary Material). The Medical Ethics Committee of the Netherlands Organisation for Applied Scientific Research TNO determined that no ethical approval was required for this study.

### Participants

We recruited reintegration professionals. In the Netherlands, there is no specific training required to become a reintegration professional working at a municipality. Most reintegration professionals have a background (i.e., study and/or work experience) in social work. The professionals were recruited using two strategies. In the first strategy we approached managers of the municipalities with whom we have already cooperated in projects to optimise the support of professionals. We asked the managers if we could interview their professionals. After receiving the managers’ consent, we asked the managers to distribute information leaflets explaining the aim and content of the study among the professionals. Professionals who indicated an interest in participating, were invited to send an email to the primary researcher (MV), providing their name, contact information and the municipality they work. In the second strategy we informed professionals at a national conference, to which professionals of all municipalities were invited, of the possibility of participating in the study. They too could apply by providing their name, contact information and the municipality they work for in an e-mail to the primary researcher (MV), after which they were sent an information leaflet. We selected professionals using consecutive- and voluntary sampling [21], including professionals who are Dutch-speaking, had over 5 years of experience and provided current and frequent support to clients to facilitate their RTW. We also selected professionals from four different municipalities, since each municipality has its own approach to work participation. In two municipalities, the execution of the legal aspect and support to RTW was performed by two separate professionals, in which we spoke to the person facilitating the support to RTW. In the other two municipalities, these functions were performed by one professional. Municipalities subdivide clients (i.e. people who receive a disability benefit and are supported towards RTW) into client profiles, categorised according to the estimated time to RTW or how ‘work fit’ a client is. We took care to select professionals supporting clients of all client groups; people who were not ‘work fit’ and had an estimated time to RTW of over two years, ranging to people who were work fit and were estimated that they could RTW right away.

### Data collection

The semi-structured interviews with professionals were conducted by telephone in November 2018 and had a duration of an hour on average. The interviews with the professionals were conducted by an experienced female researcher (MV, PhD) working in the occupational health field. Participants were informed that all information obtained prior or during the study would be handled confidentially and that an audio recording would be made. Prior to the interview, all participants were asked to provide a verbal consent which was audio recorded. During the semi-structured interviews with the professionals, we zoomed in on the different steps supporting SDM in both their *current* interaction with their clients and their *ideal* interaction with their clients, specifically team talk (having a safe relationship and the professional and client collaborating as a team, informing clients that they can be part of the decision-making process and discussing shared goals), option talk (informing clients of all available options, and the pros and cons of these options), and decision talk (discussing the preferences of both the professional and the client of the available options and making a shared decision). We also focused on the execution and evaluation of the decisions and preconditions of the use of SDM. We interviewed participants until data saturation was reached.

### Data analysis

The audio recordings of the interviews were transcribed verbatim. The transcripts were coded according to content analysis, applying open and axial coding [22], using the Atlas.ti software program. Themes were derived from the interview guide, following the three-talk model of Elwyn et al. [18]. The researchers MV and MaVi coded one of the transcripts independently using open coding, after which they discussed the codes until they reached a consensus. MaVi then coded the remaining transcripts. Afterwards the retrieved open codes were categorised into subjects and themes. The themes are described in the results section. During the process, the list of open, axial and selective codes was repeatedly checked by the primary researcher (MV) and discussed with the entire team to check the codes and reach a consensus.

## Results

We conducted a total of fourteen interviews with reintegration professionals from four different municipalities. in our analysis, we found 19 subjects divided in seven themes. We included quotes of the participating professionals to illustrate our findings.

### Team talk

#### Professional and client collaborating as a team

Most professionals roughly follow the same procedure for new clients: first the intake, then guiding the client towards being ‘work fit’, after which a client can RTW. The intake is where the professional and the client first meet and an overview of the client’s situation is established. Professionals all emphasised, especially in this phase of the process, the importance of building an alliance with the client. One professional explained this by stating that “you have to build trust between each other”. Professionals indicated that building a relationship and trusting each other is the foundation for support towards work participation. Building a relationship takes time, but according to some professionals investing time in a relationship from the start, will pay off later.

*“In any case it is important to build a relationship with clients. It doesn’t even have to be because you want them to get a job, but this way you are in touch more often. I think that helps.” – Reintegration professional (8).*

A good relationship and trusting each other is indicated as a precondition for clients opening up about their lives and” getting through” to the client, and have him/her provide a professional with information and insights into the client’s behaviour. By “getting through to the client” some professionals meant: teaching clients to reflect on their actions towards work participation and on their wishes and preferences, and also, according to some professionals, to gain a feasible perspective on work participation. Gaining insight into a client’s situation and learning about the obstacles a client faces on the road to work participation, are important in making a good diagnosis and planning follow-up actions. Professionals also indicated that a good relationship increases both the motivation of clients toward RTW and a willingness to take action to reach that goal.

*“Interviewer: So in fact you sort of guide people toward gaining insight? Interviewee: Yes, that is right. It takes up a lot of time, you know? At least three hours of work, and more often than not they do not enjoy it much, because I ask them a lot of questions. And they think, yes well, shouldn’t we get started already (with actions to RTW)? I often have to explain, yes, but I have to know more about you before I can really start helping you. OK, in the end they get it, but they do not like it, because they want this problem solved as quickly as possible.” – Reintegration professional (14).*

Professionals explained that they build relationships with clients by introducing themselves, using small talk, by making jokes and having a laugh, being informal in their one-on-one communication and by having informal contact moments by app or e-mail to foster the relationship. Some professionals indicated that to establish a good relationship you must approach a client as an equal partner. They label the relationship with their clients as “being a team” in which “you must have the same goal in mind”. One professional stated that “to be able to do this work you have to be empathetic”. All professionals stated that building a relationship is compatible with their ideal interaction with clients.

*“I feel it is important to make contact first, to start a relationship with clients. Because you have to earn their trust, and vice-versa. I see it as a commitment, just like a marriage; it does not happen overnight. You have to get to know them, and once that is sorted, it becomes easy.” – Reintegration professional (6).*

#### Explaining to clients that they can be part of the process

None of the interviewed professionals indicated that they informed their clients that they can be part of the decision-making process or recognised the need for this in their ideal interaction with clients. Most of the professionals interpreted this question differently. For instance, professionals responded that they explain their role as a professional to clients, and inform clients about their legal obligations (e.g., clients must cooperate and accept work offers whenever they can in order to receive their monthly disability benefit). Some professionals even interpreted the question of informing their clients, as informing other professionals about collaboration instead of collaborating with the client.

*“In any case I also try to explain my role in this, and the reason it is important and why, what I inquire from supervisors of clients and that, when it involves benefits, they (clients) must know that they have rights of course, but obligations as well, and what those obligations are, and if that is something the client can accept. Because I really want all that to be clear (to the client).*” – *Reintegration professional (7).*

#### Setting a shared goal

All professionals indicated that the goal of their support is the client’s RTW, as instructed by most municipalities. Professionals indicated that they strive to make clients ‘work fit’, meaning that a client is ready to apply for a job, followed by RTW. To achieve this goal, professionals explained that they use a step-by-step approach. They seem to formulate these steps themselves, but the steps are not written down or shared with the client, which limits SDM. Some professionals interpreted ‘setting a shared goal together’ as ‘setting a shared goal with colleagues’.

*“I also often consult with other professionals. We will get together and sometimes we will decide what to do with a client and how we can approach it together.” – Reintegration professional (8).*

However, all professionals clearly endorsed the importance of involving the client in setting a shared goal and aim to align their approach with the client’s preferences. Some professionals were motivated by the idea that the client is held accountable and should act on this responsibility. Other professionals want clients to be self-reliant, so that clients learn to have autonomy and develop self-confidence in the choices they make. Professionals promoted self-management in clients by giving them homework to think about their RTW goals, which could then be discussed in the next conversation. Many professionals explained that if they “did not tailor the job to the needs of the client, they would not obtain a long-term solution for the client”.

*“I want the candidate to take the lead, for the candidate to have self-determination and to give her a level of autonomy. So when I notice, especially in that area, that she could be a bit stronger, I will certainly try to cultivate that. So I try to encourage her to make choices. ( …*) *“I’ll say, ‘think about what you would like to do and let’s discuss it’.” – Reintegration professional (1).*

In some cases, the preferences of the client and the professional are in alignment. In other cases, clients have different goals and ideas about their future work participation, or have unrealistic goals according to the professionals. In case of the latter, professionals indicated that they encourage clients to reflect on a more feasible perspective. Another option would be that professionals suggest what they believe is a feasible alternative in line with the preferences of the client. If that method is not an option, professionals then actively ask questions to obtain more information from the client in order to suggest an alternative option.

*“Many people want to get back to work and let you know ‘I want this and that’. And that is not possible, because at this particular moment that is not feasible. Sometimes people do not understand why not. So you want to start at the bottom of that ladder. And you want to know what someone is capable of, what someone does. What situation are they in? And once you know more, you start to figure out which approach might suit someone. ( …) And slowly build to where you can say ‘why do you not go for a walk outside?’ But you can also set targets with a client. These can be baby steps, but, you know, with some people that way is the only way”.* – *Reintegration professional (10).*

Professionals reported several preconditions for setting a shared goal. Firstly, clients should have a certain level of autonomy and self-management. In other words, clients must be able to stand up for themselves and follow up on decisions. If clients are not autonomous, professionals opt for a more paternalistic style with clients, in which professionals make all the decisions. Secondly, professionals state that SDM is only possible with clients motivated toward RTW and able to follow up on decisions made. In summation, ideally a client must be assertive, show initiative and demonstrate that he or she follows up on decisions before professionals opt to apply SDM.

*“Well, when I think about working together with my candidates, I think of it as facilitating. And what that means is, let’s see what the candidate really wants and see to what extent we can accommodate him or her, and see what obstacles are blocking the way and how I can remove them. And sometimes the problem lies with the candidate who will have to do something to remove those obstacles.”* – *Reintegration professional (1).*

### Option talk

#### Presenting choice options

Professionals indicated that asking about a client’s preferences and goals, frequently results in various choice options which professionals and clients can discuss. In most cases, clients are asked to prepare a range of options before the meeting. A precondition for this discussion, according to the professionals, is for the client to provide feasible choice options. Furthermore, professionals generally did not actively present different choice options for a variety of reasons. The first reason being the physical and mental limitations of clients, making it difficult for professionals to match clients 1-on-1 with available choice options, in most cases pre-purchased interventions. Another reason, according to professionals, is that many clients lack the necessary level of autonomy and self-management to discuss choice options. However, there were also professionals who saw the possibility of presenting choice options regardless of the level of self-management.

*“Sometimes their level (of self-management) is so low that they cannot really see the consequences of their actions. That makes it more difficult to tackle this together, so I have to take more decisions. So with some (clients) you go on this journey together, and discover what can be done and which steps we need to take to get there. And with others you might just say ‘these are the two steps available to you, which one shall we pick?’ In that case, I make sure that the choices are more limited and more concrete.” – Reintegration professional (5).*

The third reason refers to the job descriptions of the professionals in the different municipalities. Some professionals explained that they “only perform intakes and subsequently decide which re-integration intervention or trajectory is better suited to the client’s profile”. Then they refer the client to a particular intervention or trajectory. Other professionals reflected on the division of goal-oriented and the legal aspects of the job; professionals who were only responsible for goal-oriented aspects of the job stated that they found it easier to build a relationship with the client. They had the idea that the client trusted them more, because they were not responsible for checking if a client is entitled to benefits.

*“That is the advantage of not having to take that decision (if a client is entitled to benefits). If I were to tell someone ‘if you do not show up, I will make sure you are penalised by a hundred per cent or I’ll have your benefit revoked’, then I am the bogeyman. They will never ever confide in me again.”* – *Reintegration professional (11).*

A fourth reason is that the municipality has pre-purchased interventions that do not match clients’ profiles and lead toward RTW. Some professionals indicated that in their ideal interaction with clients they would have more autonomy in the choice options, meaning no pre-purchased interventions and working demand-driven instead of supply-driven. As stated in team talk, professionals emphasise the importance of aligning the steps with the goals and preferences of clients. However, professionals experience this as a discrepancy between municipal policy and the support to RTW which professionals provide.

*“In the organisation they focus on what people can do physically and what their job profile is, previous work experiences, so they can be placed in a certain sector. For instance: ‘Someone is able to sit, so he or she can do production work, because that is done sitting down.’ Instead we should be looking at what someone would really like to be doing and how we can enable that. Working with those job profiles I think is the old way of working.” – Reintegration professional (1).*

Finally, some professionals mentioned that time and training for themselves and their colleagues are preconditions to present choice options. Most professionals experience a high caseload allowing limited time per client. Ideally, they prefer to have more time with clients to offer support of a higher quality and more SDM.

*“They expect our work to be tailored to clients’ needs and that is important to clients and suits them best. Within the entire official system and all its legal rules and regulations. And still you hope you help or counsel someone as best you can. Except, well, you just have to run production, let’s be honest. You just have to handle request.” – Reintegration professional (7).*

#### Discussing pros and cons

None of the professionals reported on discussing the pros and cons of choice options with the clients. However, professionals did report on having a preference for a choice option of their own, described as the” feasible perspective”. This feasible perspective is the result of an estimate made by the professional, which seemed to be primarily an unconscious process of weighing the pros and cons while searching for a feasible option for the client, preferably in line with the wishes of the client as described in team talk. Factors influencing this weighing process include the client’s preferences, obstacles the client faces (e.g. disabilities, housing conditions, presence of children), the amount of autonomy a client can handle, if the client is able to reflect on his/her behaviour, if the client shows initiative, and the client’s general and job- or sector-specific skills. The interaction with clients described above is consistent with their ideal interaction with a client.

*“What I try to do, is find out what will energise my clients, what are they passionate about. That is what I look for. For instance, that candidate would not mind being a beautician … And if you keep digging you might come up with something in a perfume and cosmetics shop where she might be able to give beauty tips to customers, that kind of thing. So pursue that and the road you have to take starts to appear, of what she might be able do over time … And that is how I keep peeling away until we come to a realistic, feasible perspective.” – Reintegration professional (1).*

### Decision talk

When we asked professionals whether they make shared decisions, most professionals stated that they currently make shared decisions with their clients during their interaction, and that these shared decisions conform to their ideal interaction with the client. However, follow-up remarks show that, although they want to involve the client in decisions, in the end they still make the decisions.

*“I try doing it with a bit of indirect communication: you want to be a good father, don’t you? Yes. Don’t you want to take care of your family? Yes. And don’t you want to build a future for yourself? Yes. I said, but then you will have to take steps to get there and one of those steps is taking good care of yourself and of course you will be a good father.” – Reintegration professional (2).*

Some professionals even clearly indicated that “they have the final say”, mostly because they find that they can make a better assessment of what is the most feasible perspective for their clients toward RTW.

*“We try as much as possible to do things in consultation. They can always come up with suggestions. But I make the decision.” – Reintegration professional (2).*

In addition, all professionals indicated that to not all clients are able to handle choices, because of their mental limitations, cultural differences, low autonomy, low self-reflection or lack of initiative.

*“Sometimes their level is so low that they cannot really see the consequences of their actions. That makes it more difficult to tackle this together, so I have to take more decisions.” – Reintegration professional (5).*

### Execution and evaluation of decisions

Some professionals explained that clients do not always follow up on decisions made in previous conversations.

*“He said: ‘I would really like to work.’ OK. In the previous session he had made a CV. I said, I did not receive your CV yet, send me your CV. ‘Yes, all right.’ I was on holiday for a week, autumn half-term, no CV. So I sent him another message. ‘Where is your CV? I have not received it yet.’ And then you get this whole story, well, my son had a fall and now he is in a cast and, well, lah-di-dah, I have not had time and do not pressure me.” – Reintegration professional (2).*

Ideally, professionals would like clients to follow up on decisions made. Furthermore, none of the professionals reported on evaluating the decisions made (together) or asking for input of the client in this evaluation.

## Discussion

In this article we explored whether and to what extent reintegration professionals used SDM steps in their interaction with clients and if they would like to use one or more steps in their ideal interaction with their clients. Results show that reintegration professionals found it very important to have a good relationship with clients, to trust each other, and to work together as a team. They did not inform their clients that they could be part of the decision-making process or discussed a shared goal. Although professionals did emphasise the importance of aligning their approach with the preferences of the client and they tried to discuss some choice options, they did not discuss all available options or the pros and cons of these options, or evaluated decisions with their clients. They also did not mention these aspects in their ideal interaction with clients. Preconditions mentioned are either connected to the client, such as having motivation and self-management, or to the organisation, such as having choice options, having a reasonable caseload to apply SDM and reflecting on having to perform both goal-oriented and legal aspect of the job.

Trust, collaborating as a team, and having an alliance are expressed by professionals as essential elements of supporting a client toward RTW in this study. These are important steps, as emphasised in SDM literature [18], and also in literature focusing on supporting clients toward RTW in general [12, 15]. Although found to be essential, de Winter et al. [23] stated that most of the municipalities in the Netherlands focus on checking entitlement to benefits, and fraud. This puts a damper on trust, collaborating as a team and having an alliance, and as a result it limits SDM and the support of clients toward RTW in general. This is emphasised by research stating that control decreases intrinsic motivation, because it fails to satisfy the basic needs of clients [24]. This means that although professionals clearly mention the necessity of building trust and collaborating together, the policy of municipalities may counteract clients’ autonomy and intrinsic motivation. Municipalities should therefore consider the effect of the legal aspect of the job, and consider whether focusing on trust and collaboration (i.e., goal-oriented activities) is more effective in achieving both SDM and the support of a client toward RTW in general.

Not all steps of SDM are performed. For a start, professionals generally do not inform their clients that they can be part of the decision-making process or provide the client information about the various choices and their pros and cons. This lack of raising awareness and providing information, limits a client’s self-management [16] and their intrinsic motivation as well [24]. Although professionals indicate self-management in clients as a precondition for the use of SDM in this study, clients are denied the opportunity to do so due to lack of information [16]; a vicious circle. Informing a client could be a crucial first step to break that circle. The medical sector offers various tools, such as decision aids, informational websites and campaigns [25] that provide clients with the necessary knowledge and skills, facilitating the self-management in clients and the opportunity to discuss options, and SDM, applicable to both professionals and clients in this sector.

With regard to making a shared decision, professionals clearly indicated the importance of aligning the approach of the professionals with the preferences of clients. Therefore, most professionals strive to ask the client for his or her preferences before making a decision. They do so because they find that essential to motivate the client to take steps toward RTW. Research underlines this by explaining that a client’s intrinsic motivation can be an important facilitator to RTW [12], which can be increased by clients experiencing autonomy and feeling connected to the process [24], and is achieved by collaborating with a client during the process. Although professionals state the importance of including a client’s preference in the decision, they do not go so far as to actually make shared decisions; they express that they have the final say in the decision-making process. We believe that by not only including a client’s preference in the decision, but actually making shared decisions, intrinsic motivation of a client will increase even more because of the increased autonomy of a client in being part of the process [24]. This is also the crucial difference with other available methods to facilitate support to RTW, such as supported employment or motivational interviewing [25, 26]. These methods strive to involve the clients in the process, but differ in that they do not explicitly offer choice and that the client is not actually part of the decision making process and facilitated to make a decision.

Several professionals explained that they feel limited in their choice and decision options due to the pre-purchased interventions of municipalities, and the municipalities’ focus on professionals supporting RTW. In addition, a lack of evidence of the effectiveness of these pre-purchased interventions [12, 27] limits the discussion about the pros and cons of the options. In fact, in the medical sector [28] the pros and cons are discussed based on scientific evidence, which facilitates choosing the appropriate option. To increase choice and decision options, municipalities can explore whether these interventions are effective and for whom and when. This enables professionals to explain evidence-based pros and cons per intervention and per step toward RTW, and be able to meet the needs of the individual client instead of applying a supply-driven approach.

Although professionals are willing and strive to implement SDM, professionals clearly state that SDM is not suitable for all clients; they state that SDM is only for clients who are responsive and motivated, show initiative and follow-up on plans, in other words: self-management. However, as discussed, professionals do not provide the information to build the knowledge and skills needed for self-management and motivation. This can lead to a self-fulfilling prophecy [16]. It implies that municipalities demand self-management in clients, but do not provide sufficient resources to achieve this. It could mean that there might be more clients who could be self-reliant and motivated if given sufficient information and opportunity [16]. Finally, SDM would actually increase motivation, since the client has more autonomy in the decision-making process. Both of these reasons are underlined by de Winter et al. [23]; they explain that how you treat the client determines how the clients will behave [29]. In addition, as some professionals indicated that SDM is possible when adapted to the level of self-management of the client, professionals could explore whether SDM or steps of SDM could be performed adapted to the level of self-management of clients.

A limitation of this study is that we performed the interviews by telephone, which minimised the information from non-verbal behaviour. However, we elected this method to make it possible for more professionals to participate in the study, considering their large caseload and limited amount of time. Another limitation is that we approached professionals in municipalities that we already facilitated to optimise support for clients in their RTW. In addition to voluntary sampling, this means that these professionals were most likely to be more motivated to provide optimal guidance, and subsequently SDM, but that they were also more likely to provide us with more information on the ideal and current use of SDM. Another limitation is that we did not include the perspectives of clients into this study, to reflect on the steps used in SDM. Future research is needed on these perspectives and if clients recognize and prefer steps of the SDM in the received support towards RTW. Future research is also needed to explore and acquire insight into the experiences of both professionals and clients in the use of SDM, to see which elements of the approach add value to both clients and professionals. Finally, although professionals state that they are willing and motivated to use SDM, increasing the knowledge and skills of professionals seems needed [30–34] to raise their awareness so they can reflect on the value of using SDM and the steps of SDM toward RTW. Also, providing clients with information seems necessary to facilitate self-management as a first step in increasing the applicability of SDM. Future research should focus on how and what knowledge and skills are needed for the use of SDM by professionals and clients.

## Conclusions

SDM has potential value in the process of RTW; professionals confirm the importance of building trust, collaborating as a team and aligning the approach with the preferences of the client. However, professionals currently perform a limited set of SDM steps. Professionals are not aware of all steps of SDM; steps which could be helpful in the process of RTW. Additional knowledge, skills and tools are needed for both reintegration professionals and municipalities so that professionals can consider and reflect on the value of using SDM, or SDM steps, in supporting RTW. Also providing clients with knowledge, skills and tools seems necessary to facilitate both self-management and SDM.

## Supplementary Information

**Additional file 1.** Interview guide.

**Additional file 2.** COREQ.

## Data Availability

The data is described in the themes, subthemes and anonymized participant quotations in this manuscript. Further data cannot be made publicly available due to The Medical Ethics Committee of the Netherlands Organisation for Applied Scientific Research TNO. Interested researchers may direct data access requests to the corresponding author.

## Abbreviations

RTW: Return to work
SDM: Shared decision making
COREQ: Consolidated criteria for reported qualitative research
