# Supplementary material for: Shared decision making from reintegration professionals’ perspectives to support return to work: a qualitative study
Source: BMC Public Health. 2021 Feb 9;21:325. doi: 10.1186/s12889-021-10365-z (PMC7874602; doi:10.1186/s12889-021-10365-z)
Supplement: Supplementary file 1 — Additional file 1. Interview guide. [file 12889_2021_10365_MOESM1_ESM.docx]

**Questionnaire professionals**

| Part 1 – Providing support and collaboration |
| --- |
| - Would you like to introduce yourself first? (e.g. function, background) - What thoughts come to mind when you think about collaboration with the client? - What do you mean by collaborating with the client? - When does collaboration work? Can you name examples?   - To what extent does this depend on "type" of client? Can you specify this?   - To what extent does the way of working influence this? Can you explain that?   - To what extent do you have influence on the collaboration? How?   - To what extent does the organization influence this? Your supervisor? Your colleagues? How? |
| Part 2 – Inventory of current and ideal collaboration |
| - What does your current collaboration with the client looks like?   - Can you give examples of this?   - If you should rate yourself on a scale of 0 to 10, (0 stands for no collaboration (i.e., disregarding client needs, not involving clients in choices, goals, etc.) and 10 represents the most complete collaboration in which you and the client fully collaborate and decide everything together), how do you score your own degree of collaboration? Why? - What does your ideal collaboration with the customer look like? What are your wishes? Why? - Do you see any development ambitions or possibilities for yourself in the collaboration with the client? |
| Part 3 – Steps shared decision making |
| - I name certain steps and I would like to discuss with you whether and how you both currently use and like to use this in supporting a client?   - Team talk     - Collaborating as a team     - Explain clients that they can be part of the process     - Setting a shared goal   - Option talk     - Presenting choice options     - Discussing pros and cons   - Decision talk   - Execution and evaluation of decisions - Are there preconditions to collaborate with the client?   - Characteristics of the intervention (i.e. collaboration) itself:     - Expected added value of this ideal collaboration between professional / client? What are the benefits for the client? What benefits does it offer you?     - What disadvantages does the intervention have? For the client? What disadvantages does it offer you?     - Do you already have some experience with this way of working? What do you think of this approach? Promising or not? Why or not?     - Does it match your current job description / output of your work?   - Characteristics of the professional:     - Can you tell me to what extent you and your colleagues need certain knowledge and skills to collaborate with the client?     - To what extent do you feel there is support among your colleagues for more cooperation? Why? - Characteristics of the client:   - Is it applicable for all clients, or dependent of client characteristics (duration unemployment, severity of the limitations (physical, psychological), practical obstacles (debt, informal care, care for the children, etc.)). Which knowledge, skills, attitude, belief in own ability is needed?   - Characteristics of the organization:     - What is needed in terms of support from the manager? Support colleagues? Caseload? Efficiency / legality? Overall support? |
